# Endogenous transplacental transmission of *Neospora caninum* during successive pregnancies across three generations of naturally infected sheep

**DOI:** 10.1186/s13567-018-0601-3

**Published:** 2018-10-17

**Authors:** Marta González-Warleta, José Antonio Castro-Hermida, Carmen Calvo, Valentín Pérez, Daniel Gutiérrez-Expósito, Javier Regidor-Cerrillo, Luis Miguel Ortega-Mora, Mercedes Mezo

**Affiliations:** 1 Laboratorio de Parasitología, Centro de Investigaciones Agrarias de Mabegondo, AGACAL, 15318 Abegondo, A Coruña Spain; 2 Instituto de Ganadería de Montaña CSIC-ULE, Departamento de Sanidad Animal, Facultad de Veterinaria, Universidad de León, Campus de Vegazana s/n, 24071 León, Spain; 3 SALUVET, Animal Health Department, Faculty of Veterinary Sciences, Complutense University of Madrid, Ciudad Universitaria s/n, 28040 Madrid, Spain

## Abstract

Endogenous transplacental transmission, which occurs during pregnancy as the result of reactivation of a latent infection in the dam, is the main mechanism of propagation of *Neospora caninum* within cattle herds. However, the importance of this propagation mechanism has not yet been evaluated in relation to ovine neosporosis. In this study, involving three generations of ewes naturally infected by *N. caninum*, we demonstrated that endogenous transplacental transmission may also be highly efficient in the ovine host since transmission of infection occurred in 96.6% of gestations and the congenital infection rate ranged between 66.7 and 93%. Nevertheless, parasite burdens decreased gradually in consecutive generations. Reactivation of latent infections had a strong impact on the pregnancy outcome, with high mortality rates recorded in the offspring of the two first generations of ewes (21.4–46.1%). Histological examination of the brain revealed that all aborted foetuses had characteristic lesions of neosporosis (necrotic glial foci) and a few parasite cysts, whereas most stillborn and newborn lambs that died shortly after birth had non-specific lesions (mild glial foci without necrosis) and parasite cysts were more frequent. Microsatellite analysis revealed scarce genetic variability in the *N. caninum* population, in accordance with a scenario in which infections were of a single origin and were exclusively maintained by clonal propagation through endogenous transplacental transmission.

## Introduction

*Neospora caninum* is a protozoan parasite (Apicomplexa: Sarcocystidae) with a facultative heteroxenous life cycle involving mainly ruminants and other ungulates as intermediate hosts and canids as definitive hosts. Infection of ruminants can occur postnatally, via ingestion of food and water contaminated with oocysts shed by a canid (horizontal transmission), or it can take place in utero by transplacental passage of tachyzoites (vertical transmission) from the dam to the foetus during gestation. Two types of transplacental infection have been described: (i) exogenous transplacental infection, which occurs when the dam becomes infected during pregnancy; and (ii) endogenous transplacental infection, which occurs after reactivation of a pre-existing chronic infection in the dam [1]. The infection is usually asymptomatic in dams, but can have devastating effects on their foetuses [2, 3].

Clinical neosporosis causing abortions and perinatal deaths has been recorded in all species of domestic ruminants. It is particularly prevalent and widely distributed in cattle, in which it is considered one of the main causes of reproductive failure [4–6], so that the pathological, immunological and epidemiological aspects of bovine neosporosis as well as its impact on livestock production have been studied in detail [2, 7–15]. By contrast, many aspects of infection in small ruminants remain largely unknown [16]. However, recent findings suggest that ovine neosporosis may be a more important cause of reproductive disorders than generally believed, at least in some geographical areas [17–21]. In a previous study, we demonstrated that *N. caninum* infection was the cause of the dramatic reduction in the reproductive performance of a sheep flock during two consecutive years [20]. The fact that no horizontal infections were detected on this farm led us to believe that endogenous transplacental transmission, until now considered irrelevant in sheep, merited reappraisal. We therefore designed a 3-year study in which the absence of horizontal infection was guaranteed, enabling us to demonstrate that endogenous transplacental transmission of *N. caninum* can take place in naturally infected sheep and may play a significant role in maintaining the infection in sheep flocks. Here, we provide information about the changes in the infection rate acquired by this route as well as its impact on the outcome of pregnancies in three generations of sheep. Genotyping of the *N. caninum* population implicated in abortion and perinatal deaths caused by this infection mechanism was also carried out.

## Materials and methods

### Animals: selection and management

**Figure 1:**
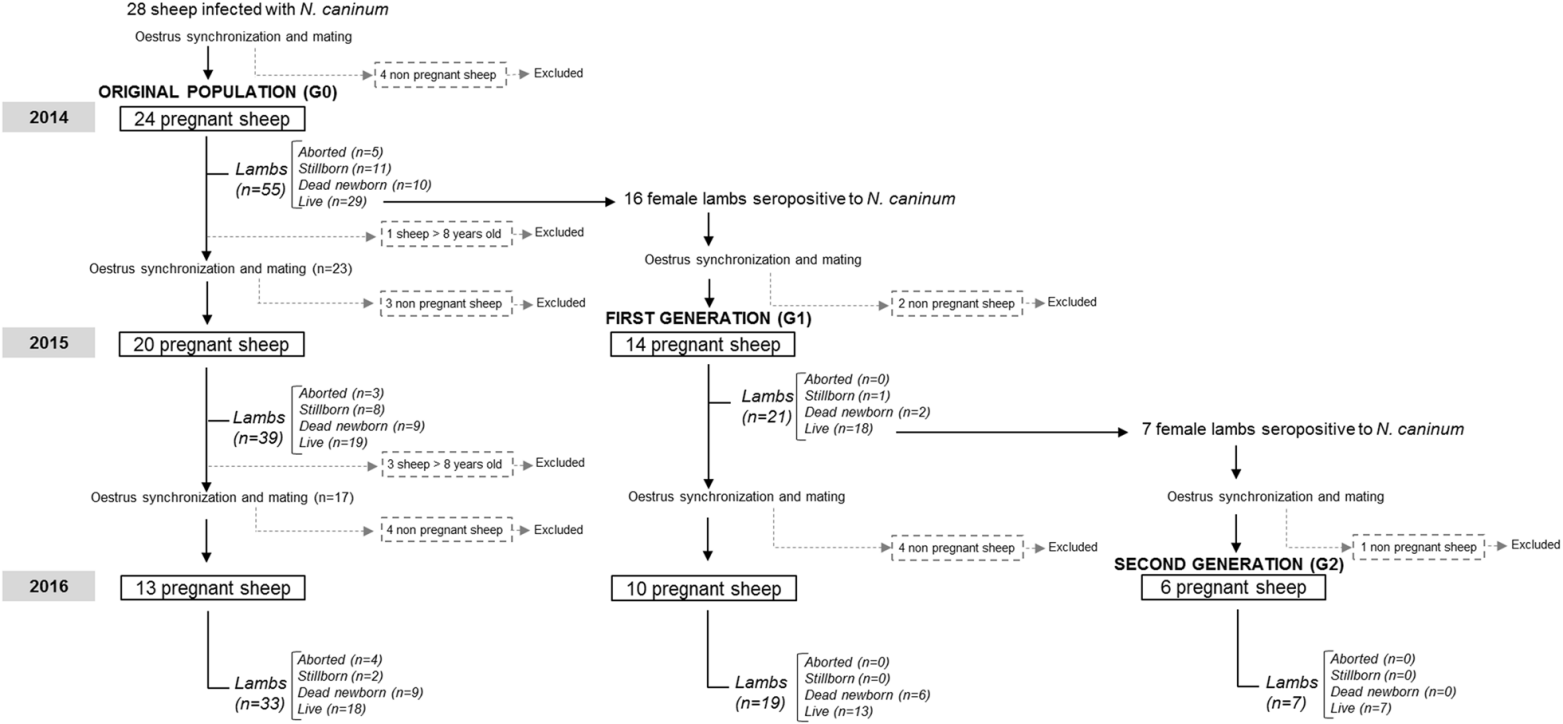
**Outline of the reproductive management and outcome of gestations for the three generations of ewes under study throughout three consecutive years (2014–2016).** The “Original population” (G0), which included 24 ewes from a commercial flock with chronical neosporosis, lambed three times. The “First generation” (G1) of ewes (*n* = 14), comprising the congenitally infected lambs born to G0 in 2014, lambed twice. The “Second generation” (G2) of ewes (*n* = 6 ), comprising the congenitally infected lambs born to G1 in 2015, lambed once.

Three consecutive generations of ewes were studied during a period of 3 years. Initially, a group of 28 Berrichon × Romanov ewes aged between 3 and 7 years was selected from a commercial flock in which *N. caninum* infection caused low fertility and a high rate of perinatal mortality [20]. These animals, categorized as the “Original population” (G0), were chosen for the following reasons: (1) all had chronic neosporosis confirmed by detection of specific antibodies in their sera and of parasite DNA in the brains of the offspring from their two last pregnancies, and (2) they were demonstrated to be seronegative to the main infectious agents causing abortion in sheep (*Toxoplasma gondii*, *Brucella melitensis*, *Coxiella burnetii*, *Chlamydia abortus*, Border disease virus and Schmallenberg virus). These 28 ewes were dewormed with albendazole (Albendex, SP Veterinaria, S.A., Tarragona, Spain) before being moved to the experimental farm facilities owned by the CIAM (Centro de Investigaciones Agrarias de Mabegondo), where they were mated. All resulting female lambs congenitally infected with *N. caninum* (i.e. with specific precolostral antibodies) were selected as “First generation” (G1) lambs. Similarly, congenitally infected female lambs produced by G1 constituted the “Second generation” (G2) (Figure 1). Throughout the study, the sheep were maintained in isolation in the CIAM facilities, so that exogenous infection by *N. caninum* was precluded. Notwithstanding, four Galician breed ewes (3 years old) from the CIAM’s *Neospora*-free flock were housed in the same facilities to act as a sentinel group for exogenous infection. All ewes and rams used for breeding were tested annually for antibodies to the aforementioned abortifacient agents.

Reproductive management of the sheep was as follows. During each year of the study, all ewes were oestrus-synchronized and then exposed for 7 days to Galician breed rams from the CIAM flock proven to be free of *N. caninum* infection. Pregnancy was diagnosed by transabdominal ultrasonography on day 45 post-mating (counting from the first day of exposure to ram). Sheep that did not become pregnant were excluded from the study, while pregnant ewes were subjected to close surveillance to record all abortions (nonviable foetuses expulsed before 140 days post-mating) and full-term births (lambs delivered from 140 days post-mating). Regarding the mortality of full-term lambs, we differentiated between stillborn lambs (prenatal death confirmed by hydrostatic pulmonary docimasy) and newborn lambs that died within 2 weeks of birth. All surviving lambs remained with their dams until weaning at age 3 months, at which time all except ewe lambs selected for further breeding were first sedated with xylazine (Rompun^®^, Bayer, Mannhein, Germany) and then euthanized with embutramide and mebezonio iodide (T61^®^; Intervet, Salamanca, Spain). Ewes older than 8 years were culled after their lambs were weaned (Figure 1).

Ewes were fed on concentrate (16% crude protein) and alfalfa hay. Different amounts of concentrate were supplied daily at the different physiological stages of the ewes, as follows: 400 g per ewe, from weaning to 30 days before mating; 750 g per ewe from day 29 pre-mating to day 100 post-mating; and 1000 g per ewe from day 101 post-mating to weaning. Alfalfa hay was supplied ad libitum. Ewes were vaccinated every year against clostridial diseases, in the last third of gestation (90 days post-mating).

### Sample collection

In each of the 3 years of the study, blood samples were collected from ewes (at mating and lambing), rams (at mating) and lambs (before first suckling). Serum samples from adult sheep were analysed fresh, while samples from lambs were stored at −20 °C until analysis. All dead offspring (aborted foetuses, stillborn lambs and lambs that died within 2 weeks of birth) were necropsied under sterile conditions to obtain samples of thoracic exudate, abomasal fluid, liver, lung, spleen, heart, kidney, semitendinosus muscle and brain. A portion of each sample was analysed fresh for microbiological isolation, and the other portion was stored frozen at −20 °C and/or fixed in 10% buffered formalin. In the lambs sacrificed after weaning, samples of semitendinosus muscle and brain were conserved frozen at −20 °C.

### Analysis for diagnosis of abortifacient agents (other than *Neospora*)

Different tests were carried out in order to rule out the presence of the infectious agents that most commonly cause abortion in sheep. Serum samples obtained from ewes and rams were screened for the presence of specific antibodies against Border disease virus, Schmallenberg virus, *C. burnetii*, *C. abortus* and *T. gondii*. For the four first infections, specific antibodies were determined using ELISA tests (IDEXX BVDV p80 Ab Test, IDEXX Schmallenberg Ab Test, IDEXX Q Fever Ab Test and IDEXX Chlamydiosis Total Ab Test; IDEXX Laboratories, Barcelona, Spain). Antibodies against *T. gondii* were determined by a latex agglutination test (Mast Toxoreagent test, Mast Group Ltd, Bootle, UK). All tests were performed following the manufacturer’s instructions.

Samples obtained during necropsy of dead lambs were analysed by different techniques [22, 23]. For the isolation and growth of *B. melitensis*, *Salmonella abortusovis* and *Listeria monocytogenes*, fresh samples of liver, lung, spleen and abomasal fluid were cultured in appropriate media. For diagnosis of *C. abortus*, impression smears from liver and abomasal fluid stained using the Ziehl–Neelsen technique were examined by microscopy. When positive results were obtained, the abomasal fluid was analysed by immune-chromatography (Clear View Chlamydia MF test, Inverness Medical, NJ, USA) for detection of bacterial antigen. Diagnosis of *C. burnetii* was based on the detection of microbial DNA in liver samples by quantitative PCR (LSI VetMAX *Coxiella burnetii* Real Time PCR kit; Life Technologies, Thermo Fisher Scientific, Delaware, USA). For detection of Border disease virus, spleen samples were analysed by quantitative reverse transcription PCR (BehiBVD/BD-VK, Vacunek S.L., Bizkaia, Spain). For diagnosis of *Leptospira* spp. and *T. gondii*, two in-house PCRs were applied to samples of kidney and brain, respectively [24, 25]. Formalin fixed samples from foetal viscera were also processed for conventional histological evaluation.

### Detection of specific IgG antibodies to *N. caninum*

For determining IgG antibody levels against *N. caninum* in ewes and rams, we used an in-house ELISA previously validated in adult sheep [20]. Briefly, the assay was carried out as follows: (1) microtitre plates were coated with 0.25 μg/well of soluble extract of tachyzoites of strain NC-1; (2) duplicate serum samples (100 μL) diluted 1:100 in phosphate buffer saline (PBS; pH = 7.4) with 0.2% Tween 20 (PBS-T) and 1% dry skimmed milk (PBS-TM) were added to the plates, which were then incubated (2 h at 37 °C); (3) plates were incubated with peroxidase conjugated anti-ovine monoclonal IgG (Sigma-Aldrich, Madrid, Spain) diluted 1:20 000 in PBS-TM (100 μL/well); (4) substrate (OPD, Sigma-Aldrich, Madrid, Spain) was added, and the optical density (OD) of the mixtures was read at 492 nm. The plates were always washed with PBS-T and all incubations were carried out at 37 °C. The same positive and negative control sera were included in each plate. The results were expressed as OD. The cut-off value for this ELISA is 0.25 (calculated from analysis of serum samples taken from 100 sheep chosen at random from neosporosis-free flocks) [20]. For lambs, the antibody response was assessed using an immunofluorescent antibody test (IFAT). Briefly, glass slides were prepared by fixing tachyzoites (10^5^/well) of strain NC-1 as previously described [26]. Precolostral sera or thoracic exudates were tested at a 1:8 dilution in PBS. The conjugate (monoclonal anti-sheep IgG labelled with fluorescein isothiocyanate, Sigma-Aldrich, Madrid, Spain) was diluted 1:500 in PBS with 0.05% Evans blue. The slides were examined under a fluorescence microscope with B-2A filters (450–520 nm). Only samples with unbroken peripheral fluorescence were considered positive.

### Detection and quantification of *N. caninum* DNA in ovine tissues

Parasite DNA was detected in samples of brain and semitendinosus muscle from foetuses and lambs. For extraction of the genomic DNA, tissue samples (20–50 mg) were treated using the commercial Realpure Genomic DNA extraction kit (Durviz S.L., Valencia, Spain) following the manufacturer’s recommendations. After adjusting the concentration of DNA to 60 ng/μL, all samples were analysed in duplicate using a nested-ITS1 PCR adapted to a single tube, as previously described [15]. Samples that tested positive by nested-ITS1 PCR were analysed by real-time quantitative PCR in order to quantify the parasite loads. This analysis included two amplification reactions, carried out in separate wells: (1) amplification of the *N. caninum* Nc-5 sequence [27] with a specifically designed primer pair [28] for parasite quantification, and (2) amplification of the ovine ß-actin gene with the previously described primer pair [29], to quantify host DNA. In both cases, the reaction mixture (total volume = 20 µL) contained Power SYBR PCR Master Mix (Thermo Fisher Scientific, Warrington, UK), the corresponding primers (20 pmol of each primer for amplification of Nc-5 sequence or 18 pmol of each primer for amplification of ß-actin gene) and 60 ng of DNA template. The amplifications were performed, under previously described conditions [28], in a Bio-Rad Multicolor Real Time PCR Detection System (Bio-Rad Laboratories, Inc., CA, USA). The number of parasites and the weight (mg) of host tissue in the samples were calculated by interpolation of Ct values from two standard curves: (1) one curve equivalent to 10^4^–10^−2^ tachyzoites with tenfold serial dilutions in a solution of ovine genomic DNA (12 ng/µL); and (2) a curve of 80–2.5 ng with two-fold serial dilutions of genomic DNA for ovine DNA quantifications. The standard curve for *N. caninum* had an average slope of −3.40 and a detection limit of 10^−1^ tachyzoites. The standard curve for ß-actin had an average slope of −3.36. The correlation coefficient for both curves was 0.99. Parasite burden was expressed as tachyzoites/mg of tissue. When the concentration of tachyzoites was not quantifiable (i.e. when it was below the detection limit of the real-time PCR), a minimum parasite burden of 10^−2^ tachyzoites was arbitrarily assigned.

### *N. caninum* genotyping

DNA samples obtained from *N. caninum* PCR-positive brains were used for genotyping by multilocus microsatellite analysis. Specifically, MS4, MS5, MS6A, MS6B, MS7, MS8, MS10, MS12 and MS21 markers were amplified using specific primers and nested-PCR conditions, as previously described [14]. For all microsatellites, the size of the PCR products was determined in a 48-capillary 3730 DNA Analyser (Applied Biosystems, Foster City, CA, USA) with GeneScan-500 (LIZ) size standards (Applied Biosystems) at the *Unidad Genómica del Parque Científico de Madrid*. The results were analysed with GeneMapper1 software, v3.5. For confirmation of allele identification, microsatellite alleles MS4, MS5, MS6A, MS7, MS10 and MS21 from representative samples were also sequenced using a Big Dye Terminator v3.1 Cycle Sequencing Kit (Applied Biosystems) and a 3730 DNA Analyser (Applied Biosystems). The sequencing was conducted at the *Unidad Genómica del Parque Cientifico de Madrid*. Sequences were analysed using BioEdit Sequence Alignment Editor v.7.0.1 software (Copyright 1997–2004 Tom Hall, Ibis Therapeutics, Carlsbad, CA, USA). Allele assignment was performed as previously described [13].

### Histology and immunohistochemistry

**Figure 2:**
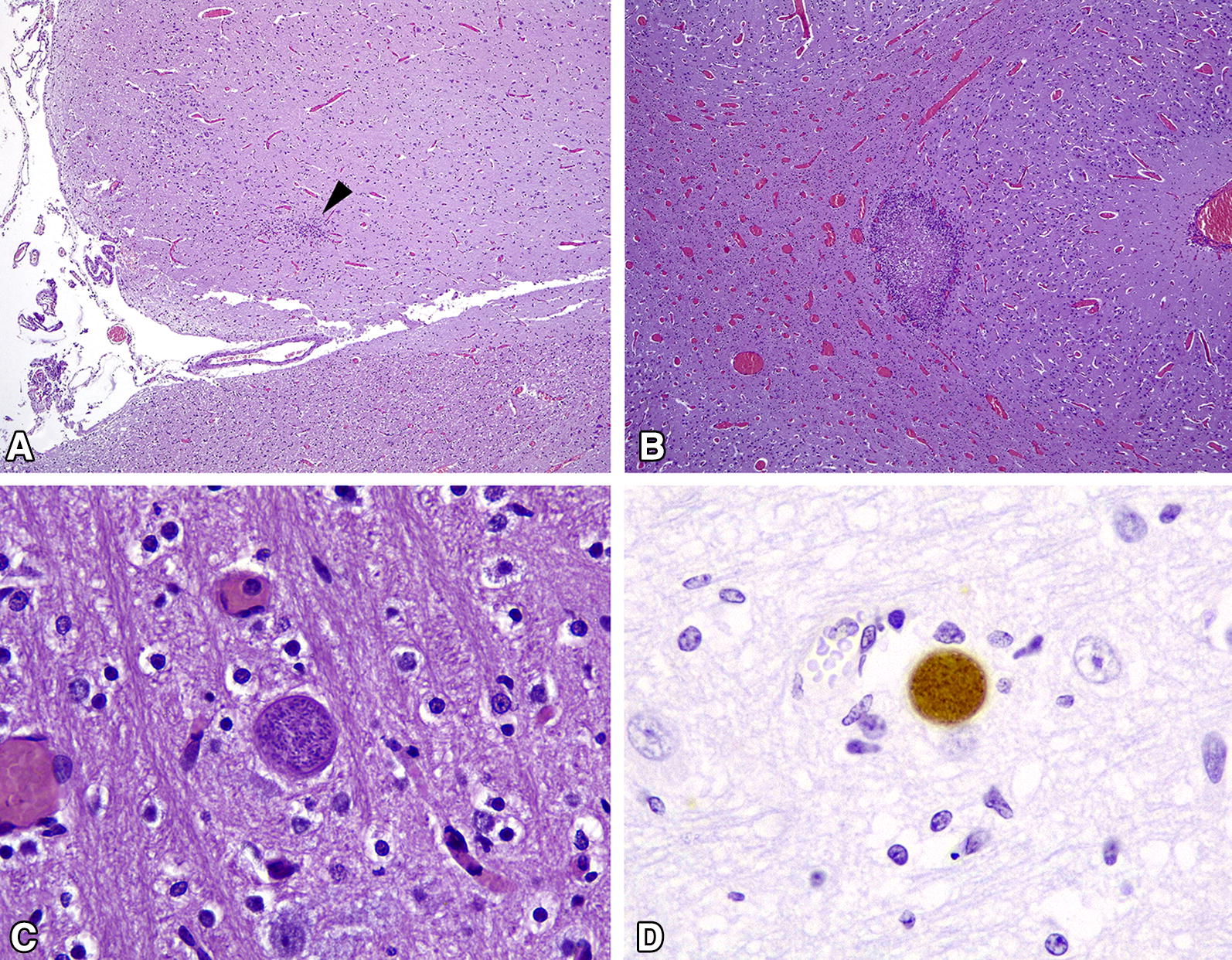
**Microscopic lesions in brain of lambs naturally infected with** ***N. caninum***. **A** Focus of gliosis (arrowhead) at the cerebral cortex. HE. 4×. **B** Diffuse congestion, mainly seen at the white matter of the *corona radiata* and a focus of necrosis with peripheral gliosis. HE. 4×. **C** Tissue cyst of *N. caninum* containing structures compatible with bradyzoites. Note the thick membrane of the cyst. HE. 40×. **D** Positive labelling of *N. caninum* tissue cyst. IHC. 40×. All pictures come from lamb offspring of G0 ewes.

Buffered formalin fixed tissue samples of aborted foetuses and dead lambs were processed for conventional histological analysis. Tissue samples were dehydrated, embedded in paraffin, cut into 4 µm-thick sections, and finally stained with haematoxylin and eosin (HE). Immunohistochemical detection of *N. caninum* antigen was carried out, according to a previously described protocol [30], on brain and semitendinosus muscle. Tissue sections were first treated with a commercial trypsin solution (Abcam, Cambridge, UK), for 15 min at 37 °C, to enhance labelling, and they were then incubated overnight with an in-house rabbit polyclonal antiserum against *N. caninum* tachyzoites [31], diluted 1:3000. A polymer-based detection system (EnVision + system Labelled Polymer-HRP anti-rabbit; Dako, Glostrup, Denmark) was applied for 30 min at room temperature, and the reaction was developed with 3, 3′-diaminobenzidine tetrahydrochloride (DAB Peroxidase Substrate Kit; Vector Laboratories, CA, USA). The sections were counterstained with Mayer’s haematoxylin. The technique was assessed by using appropriate controls (i.e. tissue sections containing cysts of *N. caninum*, *T. gondii* or *Sarcocystis* spp. and negative controls). According to the histological features, lesions were categorized as (a) non-specific, when mild glial foci without necrosis and/or diffuse congestion were observed (Figure 2A), or (b) characteristic of protozoan encephalitis when necrotic foci surrounded by microglial and mononuclear cell infiltration were observed (Figure 2B). The presence of protozoan tissue cysts in the brain was noted, when they were observed in HE stained slides (Figure 2C) or during immunohistochemical labelling (Figure 2D).

### Statistical analysis

Pearson’s Chi square and Fisher’s exact tests were used for data involving dichotomous variables, namely transplacental passage of the parasite, establishment of the infection (presence of parasite DNA), development of antibody response in lambs and offspring mortality. The Kruskal–Wallis and Mann–Whitney tests were used to compare parasite burdens. Differences were considered significantly different at *p* < 0.05. All statistical analyses were performed using SPSS v. 24.0 (Chicago, IL, USA).

## Results

### Efficacy of endogenous transplacental transmission of *N. caninum* in naturally infected sheep

The efficacy of endogenous transplacental transmission was assessed in 97 gestations, which produced 174 lambs (see Figure 1). The results for each sheep generation throughout the study period are shown in Table 1. In all 3 generations, antibody levels at mating were high (mean values between 1.598 ± 0.424 and 2.094 ± 0.318) and they increased at lambing or abortion (mean values between 1.866 ± 0.372 and 2.480 ± 0.267); these increases were statistically significant (*p* < 0.05) in G0 and G1, but not in G2. At both sampling times, ewes of the latter generation also had lower (*p* < 0.05) antibody levels than those of the two previous generations. Transplacental passage of *N. caninum* (presence of specific antibodies and/or parasite DNA in at least one lamb in the litter) was detected in most gestations (84/87). The *N. caninum* infection status of the lambs produced from gestations with transplacental transmission is shown in the right half of Table 1. Establishment of the infection (presence of parasite DNA) was significantly higher (*p* < 0.05) in the offspring of G0 (93% infected lambs) than in offspring of G1 and G2 (73% and 66.7% infected lambs, respectively). Parasite burdens were also lower in offspring of G2 (median = 2 tachyzoites/mg) than in the offspring of G0 (median = 19 tachyzoites/mg) and G1 (median = 32 tachyzoites/mg), although the differences were not statistically significant.

**Table 1:** **Specific antibody levels and efficacy of endogenous transplacental transmission in ewes with chronic neosporosis**

Generation	Ewes					Lambs infected with* N. caninum*^4^	
	Matings		Gestations		TP^3^ N	I/T^5^ (%)	Parasite burden^6^ Me (Q_25_–Q_75_)
	N	Ab level^1^ OD $\left( {{\bar{\text{X}}} \pm {\text{SD}}} \right)$	N	Ab level^2^ OD $\left( {{\bar{\text{X}}} \pm {\text{SD}}} \right)$			
G0	68	2.094 ± 0.318^a^	57	2.480 ± 0.267^a,^*	57	114/123^♦^ (93)^a^	19 (0.01–84)
G1	30	2.091 ± 0.166^a^	24	2.360 ± 0.167^a,^*	22	27/37^♦♦^ (73)^b^	32 (2–65)
G2	7	1.598 ± 0.424^b^	6	1.866 ± 0.372^b^	5	4/6 (66.7)^b^	2 (0.01–6)

^1^ Anti-*N. caninum* IgG antibodies at mating. They are expressed as optical density (OD) at 492 nm.

^2^ Anti-*N. caninum* IgG antibodies at lambing or abortion. They are expressed as optical density (OD) at 492 nm.

^3^ TP: Transplacental passage of *N. caninum* was considered to have occurred in a pregnancy when specific antibodies and / or DNA of the parasite were detected in at least one lamb in the litter.

^4^ Infection was confirmed by detection of parasite DNA. Ewe lambs maintained live for breeding were considered infected when transmission to their offspring was detected (i.e. they gave birth to some lambs with parasite DNA).

^5^ I/T: number of infected lambs/total number of lambs.

^6^ Parasite burden is expressed as number of tachyzoites per mg of tissue. Me: median; Q_25_-Q_75_: lower - upper quartiles.

^♦^ Infected lambs= 114 (102 lambs with parasite DNA + 12 ewe lambs with parasite DNA + 12 ewe lambs used for breeding that transmitted the infection in subsequent parturitions). Total lambs from gestations with TP= 123 (127 born lambs - 4 lambs which could not be analysed).

.^♦♦^ Infected lambs= 27 (22 lambs with parasite DNA + 5 ewe lambs maintained for breeding that transmitted the infection in the subsequent parturition). Total lambs from gestations with TP= 37 (38 born lambs - 1 lamb which could not be analysed).

^a,b^ Different letters within columns indicate statistically significant differences (*p *< 0.05).

* Significantly higher (*p *< 0.05) than antibody level at mating.

When offspring results were broken down by lambing year (Table 2), we observed that the percentage of lambs congenitally infected by *N. caninum* decreased progressively during successive gestations (from 98 to 81.8% and from 77.8 to 68.4% in G0 and G1, respectively), so that the prevalence of infection in the offspring of G0 ewes was significantly lower (*p* < 0.05) in their third year of parturition (2016). Parasite burdens followed a similar trend, as lambs born to G0 ewes had a significantly higher concentration of tachyzoites (*p* < 0.05) in 2014 than in 2015 and 2016. This pattern was also observed, although it was not statistically significant, in the offspring of G1, with the lambs born in the first parturition having more parasites than those born in the second lambing (median values of 50 and 18 tachyzoites/mg in 2015 and 2016 respectively). Analysis of precolostral antibodies showed that most infected lambs were able to generate an immune response to *N. caninum*. In fact, only 3 lambs were positive for DNA (2 born to G0 in 2014 and 1 born to G1 in 2015) with negative serology (Table 2). In ewes in the control group, the serology was always negative and neither precolostral antibodies nor parasite DNA were detected in their offspring.

**Table 2:** ***N. caninum*** **infection in the offspring of each generation of ewes (G0, G1 and G2)**

Lambing year	G0			G1			G2		
	I/T^1^ (%)	Parasite burden^2^ Me (Q_25_–Q_75_)	With antibody response N (%)	I/T^1^ (%)	Parasite burden^2^ Me (Q_25_-Q_75_)	With antibody response N (%)	I/T^1^ (%)	Parasite burden^2^ Me (Q_25_-Q_75_)	With antibody response N (%)
2014	50/51^♦^ (98)^a^	65 (12–134)^a^	48 (96)						
2015	37/39 (94.9)^ab^	6 (0.01–34)^b^	37 (100)	14/18^♦♦^ (77.8)	50 (16–74)	13 (92.9)			
2016	27/33 (81.8)^b^	12 (0.01–59)^b^	27 (100)	13/19 (68.4)	18 (0.01–48)	13 (100)	4/6 (66.7)	2 (0.01–6)	4 (100)

^1^ I/T: number of infected lambs/total number of lambs.

^2^ Parasite burden is expressed as number of tachyzoites per mg of tissue. Me: median; Q_25_-Q_75_: lower - upper quartiles.

^♦^ Infected lambs = 50 (38 lambs with parasite DNA + 12 ewe lambs used for breeding that transmitted the infection in subsequent parturitions). Total number of lambs from gestations with transplacental passage of *N. caninum *= 51 (55 born lambs − 4 lambs which could not be analysed).

^♦♦^ Infected lambs= 14 (9 lambs with parasite DNA + 5 breeding ewe lambs that transmitted the infection in the subsequent lambing). Total number of lambs from gestations with transplacental passage of* N. caninum*= 18 (19 born lambs - 1 lamb which could not be analysed).

^a,b ^Different letters within columns indicate statistically significant differences (*p *< 0.05).

*Neospora caninum* genotypes were determined by multilocus microsatellite analysis in PCR-positive brain samples from ewes (*n* = 5) and newly delivered lambs (abortions, stillborn lambs and lambs that died shortly after birth; *n* = 61). Complete or partial genotypes were obtained for a total of 51 (77.3%) samples (Table 3). Most samples from both ewes and lambs shared a predominant genotype (shown at the bottom of the table). More specifically, this genotype was identified in 19 of the 25 (76%) samples in which complete genotype was determined. Moreover, the remaining 6 genotypes only varied slightly from the predominant genotype, as they differed in only 1 or 2 repeat units in only 1 and/or 2 of the MS4, MS5, MS6A, MS10 and MS21 loci. On the other hand, most sibling lambs had the same genotype, and when they did not (see offspring of G0–3, G0–11 and G0–12 ewes), the genotype variant differed by only one microsatellite and it was only observed in one of the siblings. Only the offspring of G0–17 differed from this pattern as their genotypes, although incomplete, were different from the predominant one in a least two microsatellites. Nevertheless, we could not determine whether all siblings shared the same genotype variant. All of these allelic variations were confirmed by double microsatellite analysis and DNA sequencing, by the Sanger’s method. Mixed infections by different genotypes were not detected in any of the ewes or lambs.

**Table 3:** ***N. caninum*** **multilocus microsatellite genotypes identified in brain samples from ewes and lambs**

Ewe^a^	Lamb^b^	Microsatellite genotype^c^								
		MS4	MS5	MS6A	MS6B	MS7	MS8	MS10	MS12	MS21
		(AT)_x_	(TA)_x_	(TA)_x_	(AT)_x_	(TA)_x_	(AT)_x_	(ACT)_x_ − (AGA)_y_ − (TGA)_z_	(GT)_x_	(TACA)_x_
G0–1		15	16	–	–	9.1	13	6.14.9	16	6
	2014/1	15	16	15	12	9.1	13	6.14.9	16	6
G0–2	2014/1	15	16	15	12	9.1	13	6.14.9	16	6
G0–3	2014/1	15	***15*** ^d^	15	12	9.1^d^	13	6.14.9^d^	16	6 §
	2014/2	15	16	15	12	9.1	13	6.14.9	16	6
G0–4	2014/1	15	16	15	12	–	13	***6.15.9*** ^d^	16	6 §
	2014/2	15	16	15	12	9.1	13	***6.15.9*** ^d^	16	6 §
G0–5	2014/1	15	16	15	12	9.1	13	6.14.9	16	6
	2014/2	15	16	–	–	9.1	13	–	16	6
	2014/3	15	16	15	12	9.1^d^	13	6.14.9^d^	16	6
G0–6	2014/1	15	16	–	–	–	13	6.14.9	–	6
	2015/1	15	16	15	12	9.1	13	6.14.9	16	6
G0–7	2014/1	15	***15***	15	12	–	13	6.14.9	16	6 §
	2015/1	–	–	15	–	–	13	6.14.9	16	6
G0–8	2014/1	15	16	15	12	9.1	13	6.14.9	16	6
	2015/1	15	16	15	12	9.1	13	6.14.9	16	6
G0–9	2014/1	15	16	15	12	9.1	13	–	16	6
	2016/1	15	–	15	–	9.1	13	–	16	6
G0–10	2014/1	15	16	15	12	–	13	6.14.9	16	6
	2014/2	15	16	15	12	9.1	13	6.14.9	16	6
	2016/1	15	–	–	12	9.1	–	6.14.9	–	6
	2016/2	15	–	15	12	9.1	13	6.14.9	16	6
G0–11	2014/1	15	16	15	12	9.1	13	6.14.9	16	6
	2014/2	15	16	***13*** ^d^	12	9.1	13	6.14.9	16	6 §
	2015/1	15	16	15	12	9.1	13	6.14.9	16	6
	2016/1	–	–	–	–	–	13	6.14.9	16	–
G0–12	2014/1	15	16	15	12	9.1	13	6.14.9	16	6
	2014/2	15	16	15	12	9.1	13	6.14.9	16	6
	2014/3	15	16	15	12	9.1^d^	13	***6.13.9*** ^d^	16	6 §
	2014/4	15	16	15	12	9.1	13	6.14.9	16	6
	2015/1	15	16	15	12	9.1	13	6.14.9	16	6
	2016/1	15	–	–	12	9.1	13	6.14.9	16	6
G0–13	2015/1	15	16	15	12	9.1	13	***6.15.9*** ^d^	16	– §
G0–14	2015/1	–	–	–	12	–	13	6.14.9	16	–
G0–15	2015/1	15	–	–	–	9.1	–	–	–	6
	2015/2	15	16	15	12	9.1	13	6.14.9	16	6
G0–16	2016/1	15	–	–	–	9.1	13	6.14.9	16	6
G0–17	2016/1	–	–	–	–	–	13	***6.13.9*** ^d^	16	6 §
	2016/2	***13*** ^d^	–	–	–	9.1	–	***6.13.9*** ^d^	16	6 §
	2016/3	–	–	***16*** ^d^	12	–	13	***6.13.9*** ^d^	16	– §
G1–29		15	–	–	–	9.1	13	6.14.9	16	6
	2015/1	15	16	15	12	9.1	13	6.14.9	16	6
G1–30		–	–	–	–	–	13	6.14.9	16	–
	2016/1	15	16	–	–	–	13	6.14.9	16	6
G1–31	2015/1	–	–	–	–	9.1	–	6.14.9	16	6
G1–32	2016/1	15	16	15	12	9.1	13	6.14.9	16	***5***^d^ §
G1–33	2016/1	***14*** ^d^	16	15	12	9.1	13	6.14.9	16	6 §
G1–34	2016/1	15	***15*** ^d^	15	12	–	13	6.14.9^d^	16	6 §
G1–35	2016/1	15	16	15	12	9.1	13	6.14.9	16	6
G2–53		15	16	15	12	9.1	13	6.14.9	16	6
G2–54		15	16	–	12	9.1	13	6.14.9	16	6
Predominant genotype		15	16	15	12	9.1	13	6.14.9	16	6

^a ^Ewes are identified with a code indicating “Generation-ewe number”.

^b ^Lambs are identified with a code indicating “Birth year/lamb number”.

^c ^Microsatellite alleles are expressed as the number of repeats (x, y and z) in the motif sequences according to fragment size analysis and sequencing [14].

^d ^Alleles sequenced by Sanger method to check repetitive motives.

§ Genotypes differing from the predominant genotype; the alleles involved are highlighted in bolditalic type.

### Impact of reactivation of latent *N. caninum* infections on pregnancy outcome

In most pregnant ewes in this study, a reactivation of the latent *N. caninum* infection occurred with the subsequent transplacental passage of the parasite. Notwithstanding, most of these gestations came to full term, as only 6 out of 84 (7.1%) failed, with 9 foetuses aborted between days 87 and 130 after mating (ewes of G0; Table 4). Nevertheless, many G0 ewes gave birth to stillborn or weak lambs, which had to be helped with first suckling and perished within 2 weeks of birth. The mortality rate among the offspring was therefore very high in the three lambings of G0 ewes (44.4–50%). In G1 and G2 ewes, there were no abortions and only one lamb was born dead. However, G1 ewes delivered a high proportion of weak lambs that died within 2 weeks of birth, so that mortality rate of the offspring remained high (21.4%–46.1%), with no significant differences relative to G0. All infected offspring of G2 survived until weaning; however, the number of lambs in this group was very small, so that statistical analysis of the data was not possible. The four ewes in the control group had a total of 12 gestations over the 3 years study period, giving birth to 22 lambs, 21 of which lived until weaning (data not shown).

**Table 4:** **Infected offspring**

	Aborted foetuses^a^ (N)	Stillborn lambs^b^ (N)	Dead newborn lambs^c^ (N)	Weaned lambs (N)	Mortality (%)
G0					
2014	5	10	10	25	50
2015	3	6	9	19	48.6
2016	1	2	9	15	44.4
G1					
2015	–	1	2	11	21.4
2016	–	–	6	7	46.1
G2					
2016	–	–	–	4	0

^a^Gestational age: 108 ± 14 days (87–130).

^b^Gestational age: 145 ± 5 days (140–154).

^c^Days of live: 4.4 ± 4 (1–15).

Histological lesions, either non-specific or characteristic for protozoan encephalitis, were observed in 77.5% of aborted foetuses, stillborn lambs or lambs that died shortly after birth. The proportions of infected lambs with non-specific or characteristic lesions in the brain (see Figure 2) are shown in Table 5. Characteristic lesions were present in all aborted foetuses (100%), but were scarce in stillborn and dead newborn lambs (14% and 8%, respectively), which mainly presented non-specific lesions (found in 66% and 47%, respectively). Many dead newborn lambs did not have any lesions (45%). Identification of tissue cysts in the slides was more frequent in stillborn and dead newborn lambs (62% and 69% respectively) than in aborted foetuses (18%). Apart from lesions in the brain, there were no other histopathological lesions in the other organs examined. Histopathological examination of semitendinosus muscle did not show evident lesions, even though this is a usual site of establishment of *N. caninum*, as demonstrated by detection of both DNA and antigen of the parasite by respectively PCR and immunohistochemistry. Finally, all ewes and lambs from the control group tested negative for any other abortifacient agents analysed. In the dead lamb from this group, no histological changes were observed and the parasite was not detected by immunohistochemistry.

**Table 5:** **Percentage of infected lambs presenting brain lesions and/or parasite cysts**

	Lesions			Parasite cysts (%)
	Characteristic (%)^a^	Non-specific (%)^b^	None (%)	
Aborted foetuses	100	0	0	18
Stillborn lambs	14	66	20	62
Dead newborn lambs	8	47	45	69

^a^Necrotic foci surrounded by microglial and mononuclear cell infiltration.

^b^Mild glial foci without necrosis and/or diffused congestion.

## Discussion

This is the first study to demonstrate that naturally occurring neosporosis is efficiently transmitted in successive gestations across generations of sheep through the endogenous transplacental route. Previous research in naturally infected sheep has not shed light on this aspect owing to several limitations such as the duration of the studies (only one gestation) and the impossibility of completely ruling out the horizontal transmission route [17, 21, 32]. Furthermore, the scant research carried out with experimentally infected sheep has yield discrepant findings regarding the importance of the endogenous transplacental transmission of *N. caninum* in this host species. Thus, both a high proportion (75%) [33] and a low proportion (13.9%) [34] of lambs have been reported to be infected by this route. These discrepancies may have been caused by infection with different *N. caninum* isolates (NC1 or NC-Liverpool), the dose of inoculum used (1 × 10^7^ or 1.7 × 10^5^ tachyzoites) or the route of administration (subcutaneous or intravenous), among other factors. Moreover, the study conditions are also unlikely to reflect what occurs in the field.

Endogenous transplacental transmission is the main mechanism responsible for perpetuation of neosporosis in cattle herds because of two different factors. On one hand, the transmission is highly efficient, so that most chronically infected cows give birth to infected calves. On the other hand, neosporosis rarely causes abortion or clinical signs in offspring, so that the infected female calves are often reared for breeding and continue to transmit the infection during future pregnancies [4, 5, 35]. Both of these aspects have been addressed in this study in order to assess the importance of this route in the transmission of natural ovine neosporosis. The rates of congenital infection (referred to as percentage of lambs with *N. caninum* DNA) recorded in the three generations of sheep (66.7–93%) are within the range of those reported in cattle with naturally occurring neosporosis (40.7–100%) [2, 5, 36], demonstrating that endogenous transplacental transmission may be also highly efficient in the ovine host. Consequently, neosporosis can remain for long periods of time in flocks, even in the absence of exposure to *N. caninum* oocysts. Notwithstanding, we also demonstrated that the transmission efficiency decreases gradually over gestations in the same animal but also over generations, as also observed in bovine neosporosis [11, 37] and that it has been attributed to the capacity of cows to develop a certain degree of immunity which would reduce parasite transmission. Most of the data on neosporosis transmission in naturally infected cattle have been obtained by comparison of seroprevalence in dams and their progeny. Nevertheless, detection of antibodies may not reflect the actual status and/or the evolution of the infection. In this respect, our study enabled us to confirm a significant reduction in parasite burdens of the lambs delivered by the third generation dams.

Microsatellite analysis enabled genetic characterization of the *N. caninum* population causing abortions and perinatal deaths in this study. As expected, scarce genetic variability in the circulating parasite population was observed, in accordance with a scenario in which infections had a single origin and were maintained exclusively by clonal propagation through endogenous transplacental transmission. In this respect, the predominant genotype and the variant in the MS5 locus identified in this study are the same as those previously found in the original herd from which the experimental sheep (G0) were obtained [20]. Allele variations in the less frequent genotypes would occur from the predominant genotype through the successive addition or subtraction of one or two repeat units. This allelic variation resembles the mutation pattern previously associated with these markers on the basis of the stepwise mutation model [14]. The stability of these microsatellites was therefore established in vitro, so that the observed variations could be attributed to spontaneous shifts during the transmission and spread of *N. caninum* in vivo.

During assessment of the reproductive performance of the sheep, we observed that chronic latent neosporosis did not impair fertility, as in all three generations of ewes, the pregnancy rates (percentage of pregnant ewes relative to those exposed to the ram) remained within the range of those recorded in the *Neospora*-free flocks in this region (80–86%). These observations are consistent with those reported for naturally infected cows [38, 39]. By contrast, the spontaneous reactivation of the infection during gestation had a dramatic impact on the conceptus viability, and high rates of perinatal mortality were registered over two generations. Although reactivation and transplacental transmission of the infection also occurred during gestations in the third generation of ewes (G2), the congenitally infected lambs lived until weaning. The absence of deleterious effects in these lambs was probably due to the fact that they harboured very low parasite burdens (median = 2 tachyzoites/mg of tissue). However, this could not be definitely demonstrated, because of the small number of animals in this group. Nevertheless, it has been demonstrated in sheep experimentally infected with *N. caninum* at mid gestation that the severity of the lesions caused in placenta and/or foetus as well as the course of the clinical disease are closely related to the parasite burden [40]. Judging by the evolution of parasite burdens, our study demonstrates that *N. caninum* infection may be gradually cleared over successive gestations and generations. It is tempting to hypothesize that such clearance is related to the gradual development of an effective immune response by the sheep that could help to limit the transmission of the parasite and progression of the infection. As no similar studies regarding endogenous transplacental transmission in natural ovine neosporosis have been conducted, we do not know whether the clinical course described in this work constitutes a specific pattern for the ovine host. Notwithstanding, the results of the present study clearly differ from those reported for natural bovine neosporosis, where the recrudescence of infection does not disturb gestation and most of their calves are born healthy, although congenitally infected, with survival rates of 83–100% [7, 41]. The absence of clinical signs in bovine natural persistent neosporosis has been associated with the fact that spontaneous recrudescence of *N. caninum* and the subsequent infection of the calves occur in the second half of gestation (between 20 and 33 weeks) [7, 11]. In the present study, congenital infections also occurred in the second half of gestation as inferred from the fact that most of the lambs (142/145) had specific precolostral antibodies, which are not produced at an earlier gestational age. Indeed, it has been demonstrated that the adequate functional and structural development of lymphoid tissues is reached by around 75 days of gestation [42–44]. Nevertheless, as previously mentioned, most of the lambs in our study were stillborn or died shortly after birth. These clinical findings are consistent with those recorded in ewes experimentally infected with *N. caninum* tachyzoites injected during mid (90 days) and late gestation (120 days) [40, 45], thereby suggesting that the effects of neosporosis are more damaging to sheep than to cattle, even though transplacental passage of the parasite occurs when the foetal lambs have already developed immunocompetency.

*Neospora caninum* infection in lambs was characterized by the presence of low numbers of parasites, frequently at their cyst stage and mainly located in the brain and striated muscle, apparently confirming that both tissues are key sites for long-term persistence of *N. caninum*. In this respect, *T. gondii* has been demonstrated to sense some environmental factors in brain and muscle that induce the conversion of proliferative tachyzoites to quiescent encysted bradyzoites that avoided the host immune response and persisted within the long-lived post-mitotic cells present in both tissues [46, 47]. Histological analysis revealed that aborted foetuses had a high proportion of characteristic lesions of protozoan encephalitis (i.e. necrotic glial foci) and some tissue cysts, while the stillborn lambs and lambs that died shortly after birth presented milder lesions, but more parasite cysts. This suggests that the infection was better controlled, to a certain extent, in stillborn and newborn lambs than in aborted foetuses. In the former, the larger number of tissue cysts and milder lesions are consistent with an immunologically controlled infection, so that host immune response would be less aggressive, thereby allowing the parasite to reach a quiescent stage. In aborted foetuses, a more aggressive immune response would have developed and may have been effective in avoiding the formation of tissue cyst while also causing more severe lesions that may somehow compromise the survival of the foetus. Consequently, perinatal deaths in this study would not be directly affected by growth and multiplication of the parasite in the foetal tissues. Analysis of the placentas would probably provide further useful information about this aspect of the disease; however, unfortunately the placentas recovered after natural lambing presented severe autolysis and could not be analysed. Damage caused by the multiplication of *N. caninum* and/or the in situ maternal immune response might disturb the functioning of the placenta, i.e. its capacity to deliver sufficient nutrients and oxygen to the growing foetus. Restriction of foetal growth during late stages of gestation may give rise to weakened lambs that do not resist birth stress or die shortly after being born. In this regard, placental deficiency is also known to impair neurological development of the lambs, so that they are born behaviourally slow and show poor homeothermy, which finally endangers their lives [48–50]. Only one death among the offspring of the control sheep was recorded, thereby ruling out other potential causes of perinatal mortality such as nutritional deficiencies and/or unsuitable management practices during pre-breeding period and gestation. However, it was not possible to determine whether the clinical pattern of infection was related to the breed.

In conclusion, our study findings demonstrate that endogenous transplacental transmission may be an important mechanism whereby naturally occurring ovine neosporosis can be widely disseminated among the offspring of various consecutive generations, thus perpetuating infection within the flocks. Notwithstanding, further evaluation of the influence of other factors, such as sheep breed and the *N. caninum* isolate, is required.
